# Adopting Self-Measured Blood Pressure Monitoring Among Underserved Communities (ASPIRE): A Pilot Randomized Controlled Trial

**DOI:** 10.1007/s11606-025-09646-9

**Published:** 2025-06-25

**Authors:** Rasha Khatib, Nicole Glowacki, Iridian Guzman, Osondi Ozoani, John Brill, Julie C. Lauffenburger, Alex Biskis, Melanie Gordon

**Affiliations:** 1 Advocate Aurora Research Institute, Advocate Health, Milwaukee, WI USA; 2 Enterprise Population Health, Advocate Health, Milwaukee, WI USA; 3 Center for Healthcare Delivery Sciences (C4HDS), Department of Medicine, Brigham and Women’s Hospital and Harvard Medical School, Boston, MA USA; 4 Division of Pharmacoepidemiology and Pharmacoeconomics, Department of Medicine, Brigham and Women’s Hospital and Harvard Medical School, Boston, MA USA; 5 Advocate Christ Medical Center, Advocate Health, Oak Lawn, IL USA

**Keywords:** Hypertension, Social needs, Implementation science, Self-blood pressure monitoring (SMBP)

## Abstract

**Background:**

Addressing barriers to self-measured blood pressure (SMBP) engagement through tailored implementation strategies is critical for improving hypertension-related outcomes.

**Objective:**

To evaluate the feasibility of implementing the ASPIRE Clinical Integration Package, a multifaceted intervention designed to support SMBP adoption and engagement in under-resourced primary care settings.

**Design:**

This randomized trial was conducted in 2024 at one large primary care clinic serving racially and ethnically diverse populations.

**Participants:**

Patients were eligible if they had hypertension, were prescribed ≥1 blood pressure-lowering medication, and presented to the clinic with an elevated blood pressure reading.

**Approach:**

Patients were randomized to receive a free SMBP device (control; *n*=25) or a free SMBP device and the ASPIRE Clinical Integration Package (intervention; *n*=25) which included 6 components; 1. Cuff sizing, 2. Training on accurate readings, 3. ASPIRE log, 4. Reminders/support for sharing readings, 5. Social needs screening, 6. Clinic workflow for SMBP documentation. The primary outcomes included feasibility metrics (referral, recruitment, and retention) and fidelity described in terms of the proportion of patients who received each of the 6 ASPIRE components. Secondary outcomes included SMBP engagement (1+ reading documented in the electronic health record) and change in systolic blood pressure.

**Key Results:**

In total, 50 patients were randomized and included in analyses. Referral (60.0%), recruitment (60.2%), and retention (90.0%) targets were met. Fidelity evaluation revealed that 100% of patients received components 1 - 4, 96% and 93% received components 5 and 6, respectively. At 6-months the difference in SMBP engagement was 52.0% (95% confidence interval [CI] 29.3%-74.7%) favoring the intervention arm, and the difference in change in systolic blood pressure was -11.9mmHg (95% CI -21.7, -2.1) favoring the intervention arm.

**Conclusions:**

The ASPIRE Clinical Integration Package demonstrates feasibility and acceptability in promoting SMBP adoption in under-resourced primary care settings. These findings lay the groundwork for a larger trial to assess effectiveness in improving hypertension control and reducing disparities.

**Trial Registration:**

ClinicalTrials.gov Identifier NCT06175793.

**Supplementary Information:**

The online version contains supplementary material available at 10.1007/s11606-025-09646-9.

## INTRODUCTION

Hypertension affects nearly 50% of U.S. adults and remains the leading modifiable risk factor for cardiovascular disease.^1^ Despite its burden, disparities in blood pressure control persist, particularly among Black, Hispanic, and Medicaid populations, who face higher rates of hypertension-related consequences.^2^

Self-measured blood pressure (SMBP) monitoring, or home blood pressure monitoring, effectively lowers systolic blood pressure when paired with clinical support and has been endorsed by clinical guidelines for its potential to improve blood pressure control and reduce cardiovascular risk.^3–7^ SMBP is especially effective when readings are shared with the care team so that medications are modified for optimal hypertension management.^7^ Despite its proven efficacy, SMBP remains underutilized, with adoption rates from U.S. surveys ranging from 30–50%.^8,9^

Barriers to SMBP adoption are multifaceted and have been reported at the patient, provider, and healthcare system levels. Limited access to validated devices, inadequate training and social needs, such as financial strains, contribute to low uptake among patients.^10–12^ Providers face barriers such as insufficient time during visits, lack of confidence in patients'ability to perform SMBP correctly, and limited guidance on incorporating SMBP data into care plans.^10,12^ At the health system level, a lack of standardized protocols, insufficient digital infrastructure to integrate readings into electronic health records and challenges in getting patients affordable devices hinder widespread implementation.^10^ To address these gaps, the ASPIRE Clinical Integration Package (Adapting Self-Measured Blood Pressure Monitoring to Reduce Health Disparities) was developed using user-centered design to support patients and care teams in underserved primary care settings.^13–16^ ASPIRE includes six components targeting patient, provider, and system barriers, delivered by an"ASPIRE coach."

In preparation for a multi-site Type II hybrid RCT, we conducted a pilot trial at a primary care site in Chicago’s Southside to assess feasibility, fidelity, and initial clinical effectiveness. This manuscript presents findings from the pilot’s quantitative data collection.

## METHODS

This pilot randomized control trial (RCT) was approved by Advocate Aurora Health Institutional Review Board (IRB protocol #00104818) and all patients provided written informed consent. The trial protocol has been previously registered (NCT, NCT06175793) and published.^16^ Analyses and reporting followed the Consolidated Standards of Reporting Trials (CONSORT) reporting guideline extension for pilot trials.^17^

### Study Design

This was a parallel, 2-group unblinded, pragmatic pilot RCT with participants allocated 1:1 to receive a free SMBP device (control arm; *n*=25) or ASPIRE support which also includes a free SMBP device (intervention; *n*=25) when eligible patients present to the primary care clinic for a visit with elevated blood pressure. Recruitment of participants occurred between January 5^th^ and May 31^st^, 2024. Patients were followed for a minimum of 6 months ending on November 30^th^, 2024.

### Patient Eligibility

Eligible patients were adults aged ≥18 years, with a documented hypertension diagnosis using the international classification of disease (ICD-10) code I10, were prescribed ≥1 blood pressure lowering medication, and had an elevated blood pressure reading during the qualifying primary care visit (systolic≥140 mmHg or diastolic blood pressure ≥90 mmHg) documented in the electronic health record (EHR). Patients were excluded if their blood pressure was controlled upon recheck or if they resided in a nursing home or received home health care.

### Randomization

The random allocation sequence was generated by the study epidemiologist using SAS (PROC PLAN for assignment of two treatments). Group assignments were sealed in opaque envelopes and opened sequentially by the research assistant to ensure allocation concealment. Randomized patients and the care team were not blinded to the treatment allocation given the pragmatic nature of the trial.

### Study Setting and Recruitment

This pilot took place at one primary care site at Advocate Health, a large integrated not-for-profit healthcare system in Illinois. The site is located on the South Side of Chicago and serves a predominantly Black patient population with a median household income of $70,806 and 10.7% (±1.9%) living below the Federal Poverty Level.^18^ Five internal medicine physicians and their residents serve at this clinic. This clinic was selected because it was part of a system initiative where SMBP devices were offered for free to patients as part of an ongoing donation. According to standard clinic practice, patients diagnosed with hypertension who present with elevated blood pressure during their visit are asked to perform home monitoring. Patients are typically provided with a paper-based log for recording their blood pressure readings. However, standard practice does not include additional SMBP support such as assistance with cuff-sizing, structured training, comprehensive instructions, or systematic follow-up. Additionally, there is no established workflow for documenting SMBP readings within the EHR, leading to inconsistent documentation—readings are variably entered in clinical notes, structured EHR fields, or not documented at all.

A trained research assistant was on site 3 days a week and randomized eligible patients visiting one of these physicians. It was up to the discretion of the physician to invite patients with an elevated blood pressure reading and hypertension medication prescription at the conclusion of their visit to participate in the study. Patients interested in SMBP were escorted to the research exam room to confirm eligibility by the research assistant who then consented and randomized eligible patients. All patients received a SMBP device (upper arm blood pressure monitor HEM-91210 T) from the research assistant after consent and randomization. Patients randomized into the intervention arm additionally received ASPIRE support.

### Intervention Arm

Patients received ASPIRE components 1–4 via the research assistant and components 5–6 via a medical assistant who was assigned as the ‘ASPIRE coach’. The ASPIRE coach was an existing medical assistant (MA) designated by clinic leadership to deliver ASPIRE components in addition to their normal workload. A 1-hour training session was conducted to review the ASPIRE components with the MA. At the qualifying visit, patients were (1) fitted with an appropriate cuff size and given a free SMBP device, (2) trained on its use, and (3) provided an ASPIRE log to record 4 daily readings (2 morning and 2 evening) for 7 days. They discussed how to share readings via the patient portal or at a follow-up visit. Additionally, (4) patients completed a short EHR-based social needs questionnaire. Between days 7 and 14, (5) the ASPIRE coach called patients to remind them to share readings and provided resources for identified social needs. (6) A workflow was created to help the coach document SMBP readings in the EHR for care team access.

### Control Arm

Patients randomized to the control arm received a free SMBP device per standard practice. There was no training provided on how to use the device and no instructions given by the research assistant on how many readings to take or when. There was no follow up call from the ASPIRE coach to remind patients to take, document, and share readings.

### Measurement Strategy

#### Feasibility and Fidelity Outcomes

As a pilot study, our trial focused on feasibility and fidelity outcomes as our primary outcomes outlined a priori using a set of progression criteria in the protocol.^16^ A full-scale RCT would be deemed feasible if (1) physicians are interested in referring their patients to SMBP measured by the proportion of eligible patients referred), (2) that 50 patients are successfully recruited within the 5-month study period (recruitment), and (3) ≥80% of consented patients have at least one documented ambulatory blood pressure reading during the 6 months follow up (retention).

Fidelity was assessed using a checklist that the ASPIRE coach completed for each patient. Each of the 6-ASPIRE components was coded as either completed or not completed by the ASPIRE coach for patients in the intervention arm. Details for each component were also systematically documented to ensure that the self-report was accurate.

#### Secondary Implementation Outcomes

Engagement with SMBP was defined as having at least one SMBP reading documented in the EHR by month 1. Documentation by months 3 and 6 were also explored. The mean number of SMBP readings (among patients who reported at least one reading) at 1, 3, and 6 months was also documented.

#### Secondary Clinical Outcomes

Clinical outcomes were assessed secondarily to inform the measurement strategy and sample size requirements for a future full-scale RCT. These outcomes included change in systolic blood pressure, blood pressure control, defined as ≤140/90 mmHg, and time to blood pressure control (among patients who reached control) at 1, 3, and 6 months. Medication intensification was defined as any increase in class or dose of one of the blood pressure-lowering medications and was evaluated at months 1, 3, and 6.

We chose to use 140/90 mmHg cutoff for blood pressure control instead of 130/80 mmHg as our target because it aligns with historical hypertension guidelines, allows for consistency with prior studies and datasets and is also the common target across healthcare systems in the U.S.^4,19,20^ To maximize generalizability and minimize disruptions to clinic workflow, we used EHR documented ambulatory blood pressure readings as they are conducted during routine care, rather than having study-specific procedures. Advocate has standard procedures for measuring blood pressure across clinics, and values are regularly captured using automated devices.^20^

#### Data Collection and Follow-up Procedures

Given the pragmatic nature of the trial, we did not collect any data beyond routinely collected EHR data for outcome assessment. Patient clinical and demographic characteristics were obtained from the EHR using the baseline visit. SMBP and ambulatory readings were extracted from the EHR. The EHR for this healthcare system has a distinct field for these two different types of blood pressure readings. Medication intensification was determined using prescriptions documented in the EHR. The dataset generated and analyzed during the current study are not available due the sensitive nature of the data (medical records).

#### Sample Size and Statistical Analysis

We aimed to recruit 50 patients into this pilot, consistent with recommendations regarding the minimal number of participants required to identify feasibility issues.^21^ Feasibility outcomes were estimated using descriptive statistics with 95% CIs. Between-group comparison of outcomes followed the intention-to-treat principle and focused on descriptive statistics and exploratory effect estimates with 95% CIs.^17^ Blood pressure outcomes were calculated using the most recent ambulatory reading documented at 1, 3, and 6 months. At 1 and 3 months we report results only among patients with ambulatory readings during each period. At 6 months, we reported results among all 50 randomized patients. Patients who did not have any readings during follow-up were included in the 6-month analysis and their blood pressure readings were imputed by carrying their baseline reading forward.

To inform the generalizability of our results, we compared the characteristics of randomly assigned patients with those of patients who did not consent to randomization using p-values. All analyses were performed using SAS software 9.4 (SAS Institute Inc, Cary, NC).

## RESULTS

**Figure 1.**
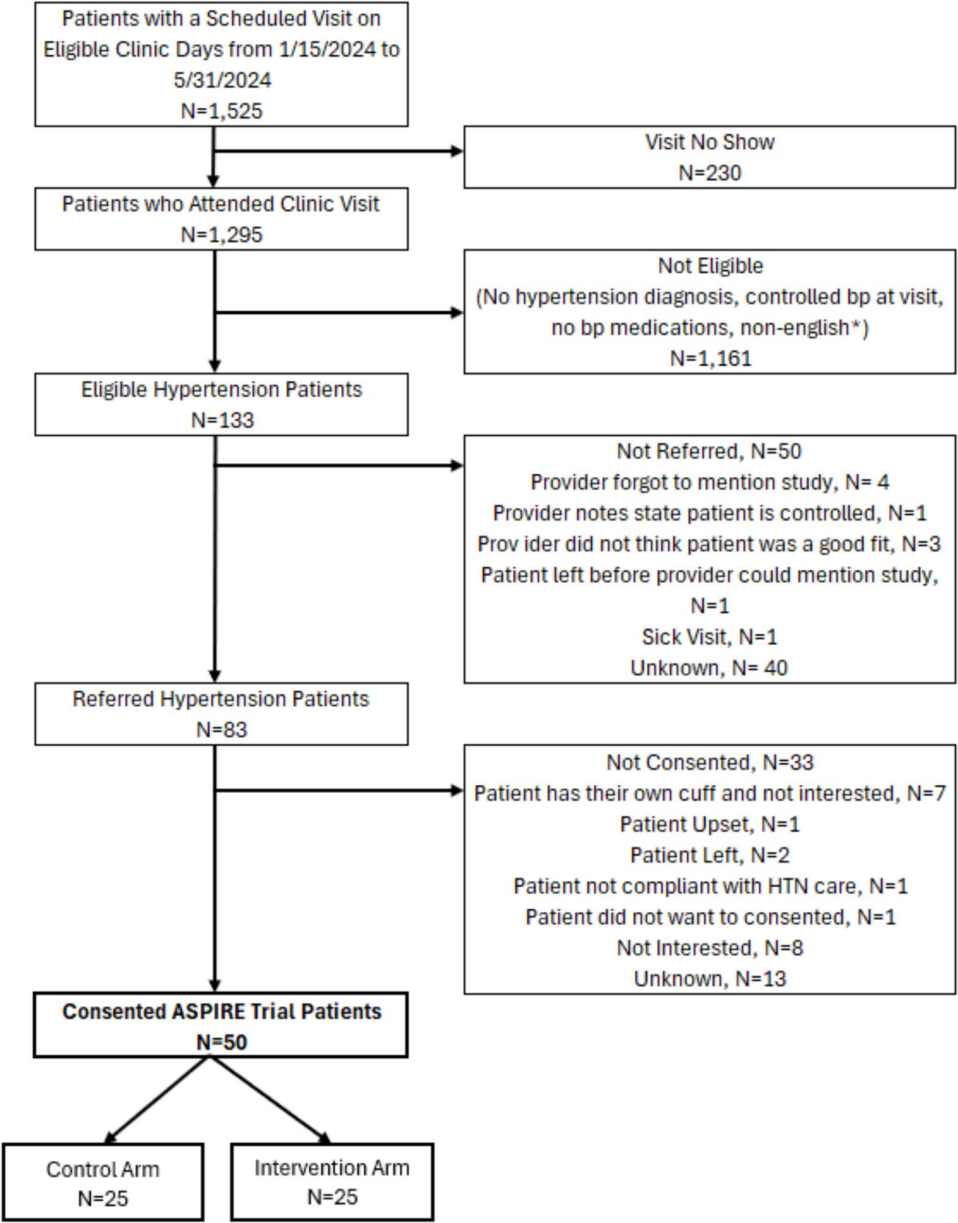
Patient flow

The CONSORT diagram is shown in Fig. 1. Patient baseline demographic and clinical characteristics of the 50 consented and randomly assigned patients are presented in Table 1. The mean (SD) age was 62.2 (13.1) years (range, 33–89 years), and most patients were female (56%), non-Hispanic Black (74%), used Medicare (50%), and preferred English (96%). At baseline the mean blood pressure was 144.4 (13.1)/82.7 (12.4) mmHg.

**Table 1:** Baseline Patient Characteristics

	Intervention	Control	Overall
	*N*=25	*N*=25	*N*=50
Age	61.5 (12.9)	62.8 (13.6)	62.2 (13.1)
<30	0 (0.0%)	0 (0.0%)	0 (0.0%)
30–49	5 (20.0%)	4 (16.0%)	9 (18.0%)
50–64	9 (36.0%)	9 (36.0%)	18 (36.0%)
65+	11 (44.0%)	12 (48.0%)	23 (26.0%)
Sex			
Female	15 (60.0%)	13 (52.0%)	28 (56.0%)
Male	10 (40.0%)	12 (48.0%)	22 (44.0%)
Race/Ethnicity			
NH-White	4 (16.0%)	4 (16.0%)	8 (16.0%)
NH-African American	20 (80.0%)	17 (68.0%)	37 (74.0%)
Hispanic	1 (4.0%)	4 (16.0%)	5 (10.0%)
Asian	0 (0.0%)	0 (0.0%)	0 (0.0%)
Other	0 (0.0%)	0 (0.0%)	0 (0.0%)
Insurance			
Commercial	6 (24.0%)	12 (48.0%)	18 (36.0%)
Medicare	12 (48.0%)	13 (52.0%)	25 (50.0%)
Medicaid	7 (28.0%)	0 (0.0%)	7 (14.0%)
Self-Pay	0 (0.0%)	0 (0.0%)	
Preferred Language			
Non-English	0 (0.0%)	2 (8.0%)	2 (4.0%)
English	25 (100.0%)	23 (92.0%)	48 (96.0%)
Comorbidities			
Current Smoker	4 (16.0%)	4 (16.0%)	8 (16.0%)
Diabetes	6 (24.0%)	9 (36.0%)	15 (30.0%)
Depression	1 (4.0%)	7 (28.0%)	8 (16.0%)
Coronary artery disease	3 (12.0%)	4 (16.0%)	7 (14.0%)
Obesity	16 (64.0%)	13 (52.0%)	29 (58.0%)
Hyperlipidemia	16 (64.0%)	13 (52.0%)	29 (58.0%)
BMI ≥30	16 (64.0%)	17 (68.0%)	33 (66.0%)
Blood Pressure at Index, mean (SD)			
Systolic Blood Pressure, mmHg	145.0 (12.0)	143.7 (14.8)	144.4 (13.3)
Diastolic Blood Pressure, mmHg	82.6 (12.0)	82.7 (13.1)	82.7 (12.4)

The characteristics of the 50 randomly assigned patients versus those who were not referred or did not consent but were otherwise eligible were similar (Appendix 1). None of the consented patients were excluded or withdrew from the study, thus all 50 patients were included in the analysis (25 intervention and 25 control patients). Appendix 2 shows that the characteristics of the 5 patients who did have any follow up ambulatory readings (11%) were similar to the 45 patients who had at least one ambulatory blood pressure reading during follow up.

### Feasibility Outcomes

Table 2 shows that the feasibility or progression criteria set for this pilot trial were fulfilled. Overall, we were able to successfully recruit 50 patients during the pre-determined recruitment period. In addition, physicians referred 62.4% (95%CI 54.2–70.6) of eligible patients, of which 60.2% (95%CI 49.7–70.8) were interested and agreed to participate. At 6 months, 90.0% (95%CI 78.2–96.7) of consented patients had at least one documented ambulatory reading during the 6-month follow-up (retention).

**Table 2:** Feasibility Outcomes

Feasibility Criteria	Outcomes of the trial
50 patients recruited within 5 months	50 eligible patients recruited and consented within < 5 months
Referral target: ≥60% of eligible patients are referred to SMBP	83/133; 62.4% (95%CI 54.2–70.6)
Recruitment target: ≥60% of referred patients consented	50/83; 60.2% (95%CI 49.7–70.8)
Retention target: ≥80% of randomized patients have ≥1 ambulatory blood pressure reading documented during 6-month follow-up	45/50; 90.0% (95%CI 78.2–96.7)

### Fidelity Outcomes

Table 3 summarizes the implementation of ASPIRE components for 25 intervention patients. All patients (100%) received the SMBP device, were measured for cuff size, and trained on device use. Most patients (88%) used the standard cuff, while 8% and 4% required small and large sizes, respectively. All patients received an ASPIRE log and indicated their preferred method for returning readings: 60% opted for in-person return, 24% via the patient portal, and 12% by phone, with 4% not specified.

**Table 3:**
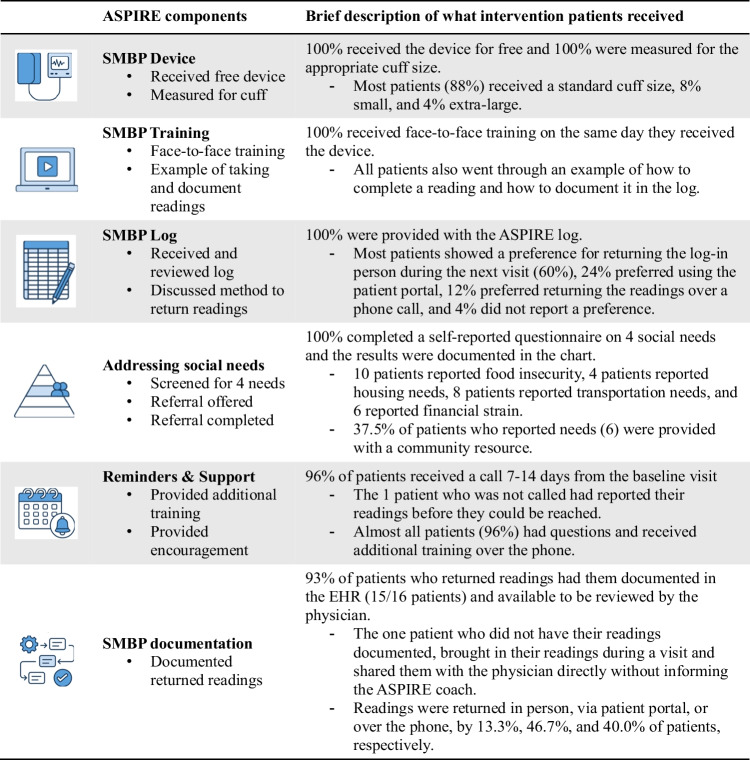
Fidelity Checklist Completed by the ASPIRE Coach on Delivering Each of the 6 Intervention Components

All patients (100%) completed a social needs survey, and 64% reported at least one need. Among them, 37.5% were offered community resources. Nearly all patients (96%) received a follow-up call within 7–14 days, except one whose readings were submitted beforehand. SMBP readings were documented in the EHR for 15 (60%) patients. Among patients with documented readings (*n*=15), they were primarily returned via the portal (47%), followed by phone (40%), and in person (13%).

### Implementation Outcomes

In the intervention arm, 15 patients (60%) had at least one documented SMBP compared to 0 patients (0%) in the control arm at 1 month. The mean number of readings in the intervention arm was 30 readings (11.2). There was no increase in the proportion of patients with SMBP readings in the intervention arm at 3 months but increased to 16 (64%) at 6 months. In the control arm, the proportion of SMBP readings increased to 2 (10%) by 3 months, and 3 (12%) by 6 months (Table 4).

**Table 4:** Implementation Outcomes and Clinical Outcomes

Timepoint	Intervention	Control	Between Group Difference (95%CI)*
	1+ SMBP readings documented- *N*(%)		
Month 1	15 (60.0%)	0 (0.0%)	60.0% (40.8–79.2)
Month 3	15 (60.0%)	2 (8.0%)	52.0% (30.1–74.0)
Month 6	16 (64.0%)	3 (12.0%)	52.0% (29.3–74.7)
	N documented SMBP readings- mean (SD)		
Month 1	30.0 (11.2)	0 (0.0)	30.0 (23.8–36.2)
Month 3	30.0 (11.2)	1.5 (0.7)	28.5 (11.2–45.8)
Month 6	28.4 (13.0)	2.0 (1.0)	26.4 (10.2–42.6)
	Change in Systolic Blood Pressure- Mean (SD)		
Month 1	−8.4	−3.5	−4.9 (−12.6- 2.8)
Month 3	−13.2	−10.3	−2.9 (−12.1- 6.3)
Month 6	−18.5	−6.6	−11.9 (−21.7- −2.1)
	Reached BP control, N (%)		
Month 1	13 (52.0%)	6 (24.0%)	28.0% (2.2–53.8%)
Month 3	19 (76.0%)	15 (60.0%)	16.0% (−9.5–41.5)
Month 6	23 (92.0%)	19 (76.0%)	16.0% (3.8–35.8)
	Time to reach blood pressure control- mean (SD)		
Month 1	21.2 (5.0)	13.1 (10.4)	8.1 (0.8–15.4)
Month 3	28.2 (12.5)	38.1 (28.3)	−9.8 (−24.6-4.9)
Month 6	43.5 (36.2)	53.9 (40.4)	−10.4 (−34.3-13.5)
	Medication Intensification, N (%)		
Month 1	6 (24.0%)	4 (16.0%)	8.0% (−14.1–30.1)
Month 3	8 (32.0%)	4 (16.0%)	16.0% (−7.3–39.3)
Month 6	9 (36.0%)	8 (32.0%)	4.0% (−22.2–30.2)

^*^ Between-group difference (95%CI) indicates the mean difference for continuous variables and the proportion difference (as a percentage) for dichotomous variables. Blood pressure results at 1 and 3 months are reported among patients who had readings during each period. At 6 months, these results are imputed by carrying the baseline reading forward

### Clinical Outcomes

The difference in systolic blood pressure between the intervention and control arm was −4.9 mmHg (95%CI: −12.6- 2.8) at month 1, −2.9 mmHg (95%CI: −12.1- 6.3) at month 2, and −11.9 mmHg (95%CI: −21.7- −2.1) at month 6. At 6 months, 92% in the intervention arm and 76% in the control arm reached blood pressure control (between group difference, 28.0% [95%CI: 2.2–53.8%]. Control was reached by 43.5 (36.2) days in the intervention arm and 53.9 (40.4) days in the control arm. Medication intensification occurred among less than half of the patients (among 36% of patients in the intervention arm and 32% of patients in the control arm; Table 4).

## DISCUSSION

Findings from this pilot RCT demonstrate the feasibility of scaling the ASPIRE Clinical Integration Package into a full-scale pragmatic trial. The package integrated effectively, particularly in a clinic serving diverse and under-resourced populations. Key feasibility metrics, including recruitment, retention, and intervention fidelity, were successfully met. However, operational challenges such as staff training, EHR workflows, and patient engagement emerged as areas needing refinement.

Our results align with existing research demonstrating the effectiveness of tailored interventions in hypertension disparities.^22^ For example, a recent study by the Johns Hopkins Center for Health Equity highlighted the impact of collaborative care models on blood pressure control among underserved populations.^23^ Initiatives such as the SMBP Implementation Toolkit developed by the National Association of Community Health Centers (NACHC), among others have sought to promote SMBP adoption through toolkits or packages similar to ASPIRE.^24–30^ However, these existing toolkits and initiatives often rely on Bluetooth-enabled or enhanced SMBP devices, which may be financially inaccessible to patients and require substantial health-IT infrastructure investments from healthcare systems. Furthermore, the real-world implementation success of these toolkits has not been systematically evaluated. ASPIRE builds on these existing initiatives and adapts some of their tools such as the AHA SMBP log and training material. Our package uses a standard SMBP device that requires minimal to no health-IT investment. It advances the field by going beyond providing a toolkit only and incorporates addressing social needs as well as a flexible and adaptable clinical workflow to document SMBP in the EHR.

The pilot achieved high intervention fidelity, consistently delivering four out of six ASPIRE components to 100% of participants. Flexible SMBP reporting methods effectively matched evolving patient preferences, shifting from initial in-person reporting to digital and telephone communication. Achieving the pre-defined referral (60%) and recruitment (62%) rates reflects adequate provider engagement and patient acceptability. However, these outcomes may partially reflect the choice of a single, highly engaged clinic for piloting. Despite universal screening for social determinants of health, participants did not utilize the offered community resources, underscoring persistent systemic barriers to accessing external support.^31–35^

Future iterations of ASPIRE will focus on sustainability by integrating the ASPIRE package with existing primary care initiatives and ensuring thorough staff training. Expanding training to all medical assistants as ASPIRE coaches, rather than relying on a single designated individual, will balance workload and enhance documentation accuracy. Additionally, adjusting the timing of reminder calls to beyond seven days may improve patient engagement. Enhanced patient engagement strategies, including refined introductory scripts and targeted educational materials, will also be developed. Ongoing qualitative research involving trial participants and providers will further inform these refinements, particularly regarding effective strategies to connect patients with necessary community resources.

### Limitations

This study has limitations. It was not statistically powered to detect differences in outcomes, so any between-group comparison should be interpreted with caution. Additionally, feasibility findings are based on experiences from one primary care site in the South Side of Chicago and may not be generalized in other contexts. Further insights from ongoing qualitative studies will enhance understanding of implementation challenges and inform preparation for a subsequent large-scale trial.

## CONCLUSIONS

Despite its proven effectiveness and endorsement in clinical practice guidelines, engagement in SMBP remains suboptimal in primary care. Findings from this pilot trial demonstrate the feasibility of conducting a full-scale trial to test and refine strategies such as the ASPIRE Clinical Integration Package aimed at improving SMBP adoption and sustained use in real-world settings.

## Supplementary Information

Below is the link to the electronic supplementary material.

Supplementary file1 (DOCX 25 KB)

## Data Availability

The dataset generated and analyzed during the current study are not available due the sensitive nature of the data (medical records)

## References

[1] **Ostchega Y, Fryar CD, Nwankwo T, Nguyen DT.** Hypertension prevalence among adults aged 18 and over: United states, 2017-2018. NCHS Data Brief. 2020:1-8 32487290

[2] **Muntner P, Hardy ST, Fine LJ, Jaeger BC, Wozniak G, Levitan EB, et al.** Trends in blood pressure control among us adults with hypertension, 1999-2000 to 2017-2018. JAMA. 2020;324:1190-1200 32902588 10.1001/jama.2020.14545 PMC7489367

[3] **Uhlig K, Patel K, Ip S, Kitsios GD, Balk EM.** Self-measured blood pressure monitoring in the management of hypertension: A systematic review and meta-analysis. Ann Intern Med. 2013;159:185-194 23922064 10.7326/0003-4819-159-3-201308060-00008

[4] **Whelton PK, Carey RM, Aronow WS, Casey DE, Jr., Collins KJ, Dennison Himmelfarb C, et al.** 2017 acc/aha/aapa/abc/acpm/ags/apha/ash/aspc/nma/pcna guideline for the prevention, detection, evaluation, and management of high blood pressure in adults: A report of the american college of cardiology/american heart association task force on clinical practice guidelines. Circulation. 2018;138:e484-e594 30354654 10.1161/CIR.0000000000000596

[5] **Muntner P, Shimbo D, Carey RM, Charleston JB, Gaillard T, Misra S, et al.** Measurement of blood pressure in humans: A scientific statement from the american heart association. Hypertension. 2019;73:e35-e66 30827125 10.1161/HYP.0000000000000087 PMC11409525

[6] **Shimbo D, Artinian NT, Basile JN, Krakoff LR, Margolis KL, Rakotz MK, et al.** Self-measured blood pressure monitoring at home: A joint policy statement from the american heart association and american medical association. Circulation. 2020;142:e42-e63 32567342 10.1161/CIR.0000000000000803

[7] **Tucker KL, Sheppard JP, Stevens R, Bosworth HB, Bove A, Bray EP, et al.** Self-monitoring of blood pressure in hypertension: A systematic review and individual patient data meta-analysis. PLoS Med. 2017;14:e1002389 28926573 10.1371/journal.pmed.1002389 PMC5604965

[8] **Poon IO, Etti N, Lal LS.** Does the use of home blood pressure monitoring vary by race, education, and income? Ethn Dis. 2010;20:2-6 20178174

[9] **Viera AJ, Cohen LW, Mitchell CM, Sloane PD.** Use of home blood pressure monitoring by hypertensive patients in primary care: Survey of a practice-based research network cohort. J Clin Hypertens (Greenwich). 2008;10:280-286 18401225 10.1111/j.1751-7176.2008.07530.x PMC8109908

[10] **Gondi S, Ellis S, Gupta M, Ellerbeck E, Richter K, Burns J, et al.** Physician perceived barriers and facilitators for self-measured blood pressure monitoring- a qualitative study. PLoS One. 2021;16:e0255578 34415946 10.1371/journal.pone.0255578 PMC8378703

[11] **Carter EJ, Moise N, Alcantara C, Sullivan AM, Kronish IM.** Patient barriers and facilitators to ambulatory and home blood pressure monitoring: A qualitative study. Am J Hypertens. 2018;31:919-927 29788130 10.1093/ajh/hpy062 PMC7190918

[12] **Wall HK, Wright JS, Jackson SL, Daussat L, Ramkissoon N, Schieb LJ, et al.** How do we jump-start self-measured blood pressure monitoring in the united states? Addressing barriers beyond the published literature. Am J Hypertens. 2022;35:244-255 35259238 10.1093/ajh/hpab170 PMC10061272

[13] **Haddad R, Badke D'Andrea C, Ricchio A, Evanoff B, Morrato EH, Parks J, et al.** Using innovation-corps (i-corps) methods to adapt a mobile health (mhealth) obesity treatment for community mental health settings. Front Digit Health. 2022;4:835002 35721796 10.3389/fdgth.2022.835002 PMC9197731

[14] **Blank s, Engel J.** The national science foundation innovation corps™ teaching handbook. Venture well. 2016.

[15] **Mora N, Arvanitakis Z, Thomas M, Kramer H, Morrato EH, Markossian TW.** Applying customer discovery method to a chronic disease self-management mobile app: Qualitative study. JMIR Form Res. 2023;7:e50334 37955947 10.2196/50334 PMC10682919

[16] **Khatib R, Glowacki N, Guzman I, Shields M, Chase J, Gordon M.** Adapting self-measured blood pressure monitoring to reduce health disparities (aspire): A pilot hybrid effectiveness‑implementation study protocol. Pilot Feasibility Stud. 2025;11:7 39815379 10.1186/s40814-024-01588-z PMC11734412

[17] **Eldridge SM, Chan CL, Campbell MJ, Bond CM, Hopewell S, Thabane L, et al.** Consort 2010 statement: Extension to randomised pilot and feasibility trials. BMJ. 2016;355:i5239 27777223 10.1136/bmj.i5239 PMC5076380

[18] Health A. Median household income, 2018-2022.

[19] **Goff DC, Jr., Lloyd-Jones DM, Bennett G, Coady S, D'Agostino RB, Gibbons R, et al.** 2013 acc/aha guideline on the assessment of cardiovascular risk: A report of the american college of cardiology/american heart association task force on practice guidelines. Circulation. 2014;129:S49-73 24222018 10.1161/01.cir.0000437741.48606.98

[20] **Lauffenburger JC, Khatib R, Siddiqi A, Albert MA, Keller PA, Samal L, et al.** Reducing ethnic and racial disparities by improving undertreatment, control, and engagement in blood pressure management with health information technology (reduce-bp) hybrid effectiveness-implementation pragmatic trial: Rationale and design. Am Heart J. 2023;255:12-21 36220355 10.1016/j.ahj.2022.10.003 PMC9742137

[21] **Sim J, Lewis M.** The size of a pilot study for a clinical trial should be calculated in relation to considerations of precision and efficiency. J Clin Epidemiol. 2012;65:301-308 22169081 10.1016/j.jclinepi.2011.07.011

[22] **Pasha M, Brewer LC, Sennhauser S, Alsawas M, Murad MH.** Health care delivery interventions for hypertension management in underserved populations in the united states: A systematic review. Hypertension. 2021;78:955-965 34397275 10.1161/HYPERTENSIONAHA.120.15946

[23] **Cooper LA, Marsteller JA, Carson KA, Dietz KB, Boonyasai RT, Alvarez C, et al.** Equitable care for hypertension: Blood pressure and patient-reported outcomes of the rich life cluster randomized trial. Circulation. 2024;150:230-242 39008556 10.1161/CIRCULATIONAHA.124.069622 PMC11254328

[24] **Meador M, Hannan J, Roy D, Whelihan K, Sasu N, Hodge H, et al.** Accelerating use of self-measured blood pressure monitoring (smbp) through clinical-community care models. J Community Health. 2021;46:127-138 32564288 10.1007/s10900-020-00858-0 PMC7755231

[25] **Fontil V, Khoong EC, Green BB, Ralston JD, Zhou C, Garcia F, et al.** Randomized trial protocol for remote monitoring for equity in advancing the control of hypertension in safety net systems (reach-sns) study. Contemp Clin Trials. 2023;126:107112 36738916 10.1016/j.cct.2023.107112 PMC10132961

[26] **McGrath D, Meador M, Wall HK, Padwal RS.** Self-measured blood pressure telemonitoring programs: A pragmatic how-to guide. Am J Hypertens. 2023;36:417-427 37140147 10.1093/ajh/hpad040 PMC10345471

[27] **Roy D, Meador M, Sasu N, Whelihan K, Lewis JH.** Are community health center patients interested in self-measured blood pressure monitoring (smbp) - and can they do it? Integr Blood Press Control. 2021;14:19-29 33603456 10.2147/IBPC.S285007 PMC7886240

[28] 29. HCCPhmhgt-pa-gh-c-pihAJ.

[29] Actions to Decrease Disparities in Risk and Engage in Shared Support for Blood Pressure Control (ADDRESS-BP) in Blacks. In: National Heart L BI, eds.; 2022.

[30] A Cardiometabolic Health Program Linked With Clinical-Community Support and Mobile Health Telemonitoring to Reduce Health Disparities. In: National Institute on Minority H HD, eds.; 2022.

[31] **Schickedanz A, Hamity C, Rogers A, Sharp AL, Jackson A.** Clinician experiences and attitudes regarding screening for social determinants of health in a large integrated health system. Med Care. 2019;57 Suppl 6 Suppl 2:S197-S201 10.1097/MLR.0000000000001051 PMC6721844 31095061

[32] **Sandhu S, Lian T, Smeltz L, Drake C, Eisenson H, Bettger JP.** Patient barriers to accessing referred resources for unmet social needs. J Am Board Fam Med. 2022;35:793-802 35896446 10.3122/jabfm.2022.04.210462

[33] **Schoenthaler AM, Gallager RP, Kaplan SA, Hopkins KA.** From screening to the receipt of services: A qualitative examination. Am J Prev Med. 2022;63:S144-S151 35987526 10.1016/j.amepre.2022.03.033

[34] **Goldstein KM, Gierisch JM, Tucker M, Williams JW, Jr., Dolor RJ, Henderson W.** Options for meaningful engagement in clinical research for busy frontline clinicians. J Gen Intern Med. 2021;36:2100-2104 33528778 10.1007/s11606-020-06587-3 PMC8298624

[35] **Tambor E, Moloney R, Greene SM.** One size does not fit all: Insights for engaging front-line clinicians in pragmatic clinical trials. Learn Health Syst. 2021;5:e10248 34667873 10.1002/lrh2.10248 PMC8512724

