# Supplementary material for: Adopting Self-Measured Blood Pressure Monitoring Among Underserved Communities (ASPIRE): A Pilot Randomized Controlled Trial
Source: J Gen Intern Med. 2025 Jun 25;40(16):3822–30. doi: 10.1007/s11606-025-09646-9 (PMC12686236; doi:10.1007/s11606-025-09646-9)
Supplement: Supplementary file 1 — Supplementary file1 (DOCX 25 KB) [file 11606_2025_9646_MOESM1_ESM.docx]

**Appendix 1**: Baseline characteristics of eligible patients who consented and did not consent to participate

|  | **Trial Patients** | **Eligible but Not Consented** | **P-Value** |
| --- | --- | --- | --- |
|  | N=50 | N=83 |  |
| **Age** | 62.2 (13.1) | 58.9 (15.3) | 0.25 |
| <30 | 0 (0.0%) | 3 (3.6%) | 0.64 |
| 30-49 | 9 (18.0%) | 18 (21.7%) |  |
| 50-64 | 18 (36.0%) | 30 (36.1%) |  |
| 65+ | 23 (26.0%) | 32 (38.6%) |  |
| **Sex** |  |  |  |
| Female | 28 (56.0%) | 46 (55.4%) | 0.63 |
| Male | 22 (44.0%) | 37 (44.6%) |  |
| **Race/Ethnicity** |  |  |  |
| NH-White | 8 (16.0%) | 11 (13.3%) | 0.19 |
| NH-African American | 37 (74.0%) | 51 (61.6%) |  |
| Hispanic | 5 (10.0%) | 17 (20.5%) |  |
| Asian | 0 (0.0%) | 2 (2.4%) |  |
| Other | 0 (0.0%) | 2 (2.4%) |  |
| **Insurance** |  |  |  |
| Commercial | 18 (36.0%) | 37 (44.6%) | 0.68 |
| Medicare | 25 (50.0%) | 29 (34.9%) |  |
| Medicaid | 7 (14.0%) | 16 (19.3%) |  |
| Self-Pay | 0 (0.0%) | 1 (1.2%) |  |
| **Preferred Language** |  |  |  |
| Non-English | 2 (4.0%) | 4 (4.8%) | 0.97 |
| English | 48 (96.0%) | 79 (95.2%) |  |
| **Comorbidities** |  |  |  |
| Current Smoker | 8 (16.0%) | 14 (16.9%) | 0.92 |
| Diabetes | 15 (30.0%) | 33 (39.8%) | 0.40 |
| Depression | 8 (16.0%) | 4 (4.8%) | 0.02 |
| Coronary artery disease | 7 (14.0%) | 13 (15.7%) | 0.67 |
| Obesity | 29 (58.0%) | 36 (43.4%) | 0.25 |
| Hyperlipidemia | 29 (58.0%) | 34 (41.0%) | 0.14 |
| BMI >30 | 33 (66.0%) | 50 (60.2%) | 0.75 |
| **Blood Pressure at Index, mmHg** |  |  |  |
| SBP | 144.4 (13.3) | 144.3 (11.9) | 0.37 |
| DBP | 82.7 (12.4) | 84.6 (10.9) | 0.31 |

| **Appendix 2**: Baseline characteristics of trial patients who have at least one ambulatory reading documented in the EHR and those who do not have readings beyond their baseline reading | | | |
| --- | --- | --- | --- |
|  | **No Follow Up** | **Follow Up** | **P-Value** |
|  | N=5 | N=45 |  |
| **Age** | 61.0 (18.9) | 62.3 (12.6) | 0.84 |
| <30 | 0 (0.0%) | 0 (0.0%) | 1 |
| 30-49 | 1 (20.0%) | 9 (17.8%) |  |
| 50-64 | 2 (40.0%) | 16 (35.6%) |  |
| 65+ | 2 (40.0%) | 21 (46.7%) |  |
| **Sex** |  |  |  |
| Female | 2 (40.0%) | 26 (57.8%) | 0.64 |
| Male | 3 (60.0%) | 19 (42.2%) |  |
| **Race/Ethnicity** |  |  |  |
| NH-White | 2 (40.0%) | 6 (13.3%) | 0.24 |
| NH-African American | 3 (60.0%) | 34 (75.6%) |  |
| Hispanic | 0 (0.0%) | 5 (11.1%) |  |
| Asian | 0 (0.0%) | 0 (0.0%) |  |
| Other | 0 (0.0%) | 0 (0.0%) |  |
| **Insurance** |  |  |  |
| Commercial | 2 (40.0%) | 16 (35.6%) | 0.83 |
| Medicare | 2 (40.0%) | 23 (51.1%) |  |
| Medicaid | 1 (20.0%) | 6 (13.3%) |  |
| Self-Pay | 0 (0.0%) | 0 (0.0%) |  |
| **Preferred Language** |  |  |  |
| Non-English | 0 (0.0%) | 2 (4.4%) | 1 |
| English | 5 (100.0%) | 43 (95.6%) |  |
| **Comorbidities** |  |  |  |
| Current Smoker | 2 (40.0%) | 6 (13.3%) | 0.18 |
| Diabetes | 0 (0.0%) | 15 (33.3%) | 0.12 |
| Depression | 1 (20.0% | 7 (15.6%) | 1 |
| Coronary artery disease | 0 (0.0%) | 7 (15.6%) | 0.34 |
| Obesity | 3 (60.0%) | 26 (57.8%) | 1 |
| Hyperlipidemia | 4 (80.0%) | 25 (55.6%) | 0.38 |
| BMI >30 | 2 (40.0%) | 31 (68.9%) | 0.32 |
| **Blood Pressure at Index, mmHg** |  |  |  |
| SBP | 149.6 (8.6) | 143.8 (13.7) | 0.36 |
| DBP | 81.6 (10.9) | 82.8 (12.7) | 0.84 |
